# Ethnic differences in COVID-19 mortality in the second and third waves of the pandemic in England during the vaccine rollout: a retrospective, population-based cohort study

**DOI:** 10.1186/s12916-022-02704-7

**Published:** 2023-01-08

**Authors:** Matthew L. Bosworth, Tamanna Ahmed, Tim Larsen, Luke Lorenzi, Jasper Morgan, Raghib Ali, Peter Goldblatt, Nazrul Islam, Kamlesh Khunti, Veena Raleigh, Daniel Ayoubkhani, Neil Bannister, Myer Glickman, Vahé Nafilyan

**Affiliations:** 1 Office for National Statistics, Health Analysis and Life Events, Newport, NP10 8XG UK; 2 MRC Epidemiology Unit, University of Cambridge, School of Clinical Medicine, Cambridge, CB2 0QQ UK; 3 UCL Institute of Health Equity, Department of Epidemiology & Public Health, University College London, London, WC1E 7HB UK; 4 Nuffield Department of Population Health, University of Oxford, Oxford, OX3 7LF UK; 5 School of Primary Care, Population Sciences and Medical Education, University of Southampton, Southampton, SO17 1BJ UK; 6 Diabetes Research Centre, University of Leicester, Leicester, LE5 4PW UK; 7 The King’s Fund, 11-13 Cavendish Square, London, W1G 0AN UK; 8 Department of Medical Statistics, London School of Hygiene & Tropical Medicine, London, WC1E 7HT UK

**Keywords:** COVID-19, Coronavirus, SARS-CoV-2, Ethnicity, Ethnic group, Mortality, Vaccination

## Abstract

**Background:**

Ethnic minority groups in England have been disproportionately affected by the COVID-19 pandemic and have lower vaccination rates than the White British population. We examined whether ethnic differences in COVID-19 mortality in England have continued since the vaccine rollout and to what extent differences in vaccination rates contributed to excess COVID-19 mortality after accounting for other risk factors.

**Methods:**

We conducted a retrospective, population-based cohort study of 28.8 million adults aged 30–100 years in England. Self-reported ethnicity was obtained from the 2011 Census. The outcome was death involving COVID-19 during the second (8 December 2020 to 12 June 2021) and third wave (13 June 2021 to 1 December 2021). We calculated hazard ratios (HRs) for death involving COVID-19, sequentially adjusting for age, residence type, geographical factors, sociodemographic characteristics, pre-pandemic health, and vaccination status.

**Results:**

Age-adjusted HRs of death involving COVID-19 were elevated for most ethnic minority groups during both waves, particularly for groups with lowest vaccination rates (Bangladeshi, Pakistani, Black African, and Black Caribbean). HRs were attenuated after adjusting for geographical factors, sociodemographic characteristics, and pre-pandemic health. Further adjusting for vaccination status substantially reduced residual HRs for Black African, Black Caribbean, and Pakistani groups in the third wave. Fully adjusted HRs only remained elevated for the Bangladeshi group (men: 2.19 [95% CI 1.72–2.78]; women: 2.12 [1.58–2.86]) and Pakistani men (1.24 [1.06–1.46]).

**Conclusions:**

Lower COVID-19 vaccination uptake in several ethnic minority groups may drive some of the differences in COVID-19 mortality compared to White British. Public health strategies to increase vaccination uptake in ethnic minority groups would help reduce inequalities in COVID-19 mortality, which have remained substantial since the start of the vaccination campaign.

**Supplementary Information:**

The online version contains supplementary material available at 10.1186/s12916-022-02704-7.

## Background

The disproportionate impact of the coronavirus pandemic on ethnic minority groups has been widely reported [1–4]. In England, rates of hospitalisation for COVID-19, admission to intensive care, and death were higher among ethnic minority groups during the first and second waves of the pandemic [5]. However, compared with the first wave, excess COVID-19 mortality was reduced in the second wave among the Black African and Black Caribbean groups but increased among the Pakistani and Bangladeshi groups [6, 7]. Moreover, adjustments for geography, socioeconomic factors, and pre-existing health conditions accounted for a large proportion of the elevated COVID-19 mortality risk observed in the first and second waves. However, some residual risk remained unexplained, most notably for South Asian and Black African groups.

The UK began its coronavirus vaccination programme on 8 December 2020, starting with those most likely to experience severe outcomes (people aged 70 years and over and those with underlying health conditions) and those working in health and social care roles [8]. Survey data indicates that rates of vaccine hesitancy in the UK are highest among people from Black ethnic groups [9]. Consistent with this, differences in vaccination rates by ethnic group were evident early in the rollout of the vaccination programme and these differences widened over time [10, 11]; by 12 December 2021, 3.7% of White British adults aged 50 or over had not received any dose of a COVID-19 vaccine, compared with 26.2% from the Black Caribbean group and 17.4% of the Black African group [12].

This study used population-level linked administrative data for England to investigate whether inequalities in deaths involving COVID-19 by ethnic group have continued into the third wave. We also explored the extent to which elevated COVID-19 mortality in some ethnic groups can be explained by differences in age, residence type, geographical factors, sociodemographic characteristics, pre-pandemic health, and, for the first time, vaccination rates.

## Methods

### Data sources

We conducted a retrospective, population-based cohort study using data from the Office for National Statistics (ONS) Public Health Data Asset (PHDA). The ONS PHDA is a linked dataset combining the 2011 Census, mortality records, the General Practice Extraction Service (GPES) Data for Pandemic Planning and Research (GDPPR), Hospital Episode Statistics (HES), and vaccination data from the National Immunisation Management System (NIMS).

Person-level datasets were created from the HES and GDPPR record-level datasets by stacking and deduplicating on NHS number and date of birth. Records with blank or invalid NHS numbers or dates of birth were dropped as these could not be linked to the 2011 Census.

### Data linkage

To obtain NHS numbers for the 2011 Census, we linked the 2011 Census to the 2011–2013 NHS Patient Registers using deterministic and probabilistic matching, with an overall linkage rate of 94.6% (see [13] for a detailed description of the linkage methodology and quality evaluation). Rates of linkage failure were higher for most ethnic minority groups than the White British group (Additional file 1: Table S1). Compared with the White British group, the unadjusted odds of linkage failure were highest for the ‘other’ ethnic group (odds ratio = 5.81, 95% confidence interval = 5.78 to 5.84), followed by the mixed ethnic group (4.44, 4.40 to 4.47) and the Chinese group (4.11, 4.07 to 4.16). Rates of linkage failure were also higher in men, younger age groups, people living in more deprived areas, and varied by region; the highest rate of linkage failure was observed for people living in London (9.2%). Once these factors were adjusted for, the odds of linkage failure in ethnic minority groups were substantially reduced. The adjusted odds ratio of linkage failure was below one for the Indian, Bangladeshi, and Pakistani ethnic groups but remained above one for the Black African (1.76, 1.74 to1.77) and the Black Caribbean (1.30, 1.28 to 1.31) ethnic groups.

Further linkage to deaths registrations data, GDPPR, HES, and NIMS data was performed deterministically based on NHS number. 86.2% of deaths recorded in deaths registrations data that occurred in England between 8 December 2020 and 1 December 2021 among people aged 30 to 100 years were included in the analysis. 79.0% of people aged 30 to 100 years who had received at least one dose of a COVID-19 vaccine during the study period according to NIMS records as of 4 January 2022 were linked to the PHDA. The unlinked deaths and vaccination records reflect people not included in our study population (see the ‘Generation of the study cohort’ section).

### Generation of the study cohort

**Fig. 1:**
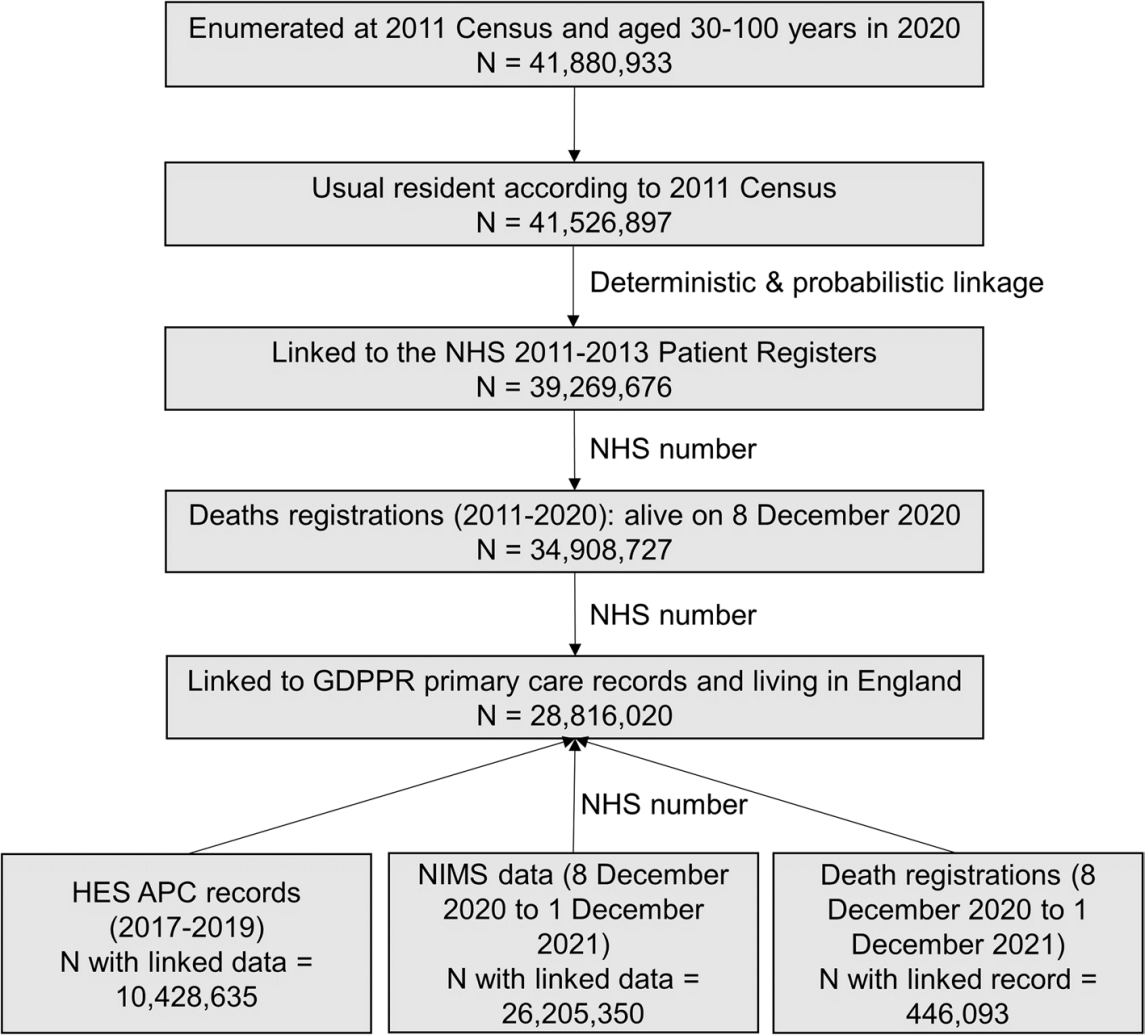
Sample selection and number of participants

Of the 41,880,933 people enumerated at the 2011 Census in England and Wales who would be aged 30–100 in 2020, we excluded 354,036 people (0.9%) who were short-term residents (i.e. people who were enumerated at the 2011 Census but did not intend on staying in the country for at least 12 months), 2,257,221 people (5.4%) who could not be linked deterministically or probabilistically to the NHS Patient register, and 4,360,949 individuals (10.4%) who had died between the Census and 8 December 2020 (the start of the vaccination campaign in the UK). An additional 6,092,707 people (14.5%) were not linked to English primary care records because they either did not live in England in 2019 (the Census included people living in England and Wales) or were not registered with a GP practice in England that was participating in GDPPR (which collects data from 6535 GP practices, covering all GP system suppliers and 97.5% of open and active practices in England [14]). The final study population included 28,816,020 people aged 30–100 years in 2020 (representing 80% of the mid-year 2020 population estimate for England) (Fig. 1) [15].

We restricted our analysis to people aged 30 to 100 in 2020 because most sociodemographic factors were drawn from the 2011 Census, which may not represent people’s circumstances at the beginning of the pandemic; younger people were thought particularly likely to have changed their circumstances, as evidenced by a greater proportion of younger people having different postcodes at the 2011 Census to the most recent postcode recorded in their GP records (Additional file 1: Fig. S1). In addition, very few deaths occurred in people aged below 30 years; official figures show that out of the 84,449 deaths involving COVID-19 in England and Wales in 2020, only 127 (0.2%) were among people less than 30 years old [16].

### Exposure

The exposure was self-reported ethnic group, retrieved from the 2011 Census. We used a 10-category ethnic group classification (White British [White English/Welsh/Scottish/Northern Irish/British], Bangladeshi, Black African, Black Caribbean, Chinese, Indian, mixed [White and Asian, White and Black African, White and Black Caribbean, and other mixed], Pakistani, White other [White Irish, White Gypsy or Irish Traveller, and other White], and other [other Asian, Arab, other Black, and any other ethnic groups]).

### Covariates

Ethnic differences in the risk of death involving COVID-19 across could be mediated by factors linked to the risk of infection (such as communal living, residing in an area with high infection rates, socioeconomic and demographic factors, and occupation) and factors associated the risk of death if infected (health status and vaccination status). These factors may fall on the causal pathway between ethnicity and COVID-19 mortality (Additional file 1: Fig. S2).

The following covariates were included from the 2011 Census data: age, residence type (private household, care home, or other communal establishment), household tenure, National Statistics Socio-economic Classification (NS-SEC), highest qualification, household size, household deprivation, family status, household composition, key worker in household, and key worker type (education and childcare, food and necessity goods, health and social care, key public services, national and local Government, public safety and national security, transport, utilities and communication; derived according to Census returns based on the 2010 Standard Occupational Classification (SOC) and the 2007 Standard Industrial Classification of Economic Activities [17]) (Additional file 1: Table S2). Body mass index (BMI; classified as underweight, normal weight, overweight, obese, unknown) and pre-existing health conditions (using the same Systematized Nomenclature of Medicine Clinical Terms [SNOMED-CT] codes as the QCovid2 risk prediction model [18]) were included as covariates from the GDPPR data. The QCovid risk prediction model was used in the UK to identify clinically extremely vulnerable individuals who should shield during the pandemic. The model has been previously shown to predict risk of COVID-19 hospitalisation and mortality in three independent datasets [19–21]. The number of admissions to, and number of days spent in, admitted patient care during the three years prior to the pandemic were included as covariates from the HES Admitted Patient Care data.

The following covariates were included from other data sources: vaccination status (from NIMS data); region and Rural Urban classification (from the National Statistics Postcode Lookup), population density of the Lower layer Super Output Area (from mid-2019 population estimates), and Index of Multiple Deprivation (IMD) (from the English Indices of Deprivation, 2019) derived from postcodes in GDPPR data; occupational exposure to disease and proximity to others for individuals and the maximum score among all individuals in each household (from the Occupational Information Network database, which collects a range of information about individuals’ working conditions and day-to-day tasks of their job. To calculate the proximity and exposure measures, the questions asked were as follows: (i) How physically close to other people are you when you perform your current job? (ii) How often does your current job require that you be exposed to diseases or infection? Scores ranging from 0 to 100 were calculated for these questions based on 2011 Census data on occupation [22]) and care-home residence status (from the 2019 NHS Patient Register).

### Outcome

The outcome was death involving COVID-19, i.e. COVID-19 International Classification of Diseases 10 code of U07.1 (COVID-19, virus identified), U07.2 (COVID-19, virus not identified), or U09.9 (post-COVID condition, unspecified) in part I or II of the death certificate, occurring between 8 December 2020 and 1 December 2021.

### Statistical analysis

Age-standardised vaccination rates by ethnic group were calculated for each wave based on the number of vaccine doses received by the end of the period (12 June 2021 for wave 2 and 1 December 2021 for wave 3). Crude rates (%) of the number of people in each ethnic group who were unvaccinated, single-vaccinated, double-vaccinated, or triple-vaccinated were age-standardised using the 2013 European Standardised Population [23].

We calculated age-standardised mortality rates (ASMRs) by ethnic group as deaths per 100,000 person-years at risk to examine the absolute risk of death involving COVID-19, standardised to the 2013 European Standardised Population [23]. ASMRs were calculated separately for each of the waves of the pandemic that occurred during the vaccine rollout (wave 2: 8 December 2020 to 12 June 2021; wave 3: 13 June 2021 to 1 December 2021). This analysis therefore excludes any deaths occurring early in the second wave, which is estimated to have started in September 2020 [24].

As the pandemic was ongoing at the end of the study period, the data were subject to right-censoring. We therefore used Cox proportional hazards models to assess whether differences in the risk of mortality involving COVID-19 by ethnic group could be accounted for by location, sociodemographic factors, pre-pandemic health, and vaccination status. Separate models were fitted for the second and third waves. The index date for start of follow-up time was 8 December 2020 for wave two (the start of the vaccination programme in the UK) and 13 June 2021 for wave three. End of follow-up was date of death for those who died or the end of the wave period for those who were still alive at the end of the period: 12 June 2021 for wave two and 1 December 2021 for wave three (see Additional file 1: Table S3 for mean follow-up times by ethnic group). For computational efficiency, we included all individuals who died of any cause during the analysis period and a random sample (selected by simple random sampling without replacement) of those who did not, with sampling rates of 1% for the White British ethnic group and 10% for every other ethnic group; case weights equal to the inverse probability of selection were included in the analysis, following previously published methods [6, 13]. The White British group was used as the reference category in all models.

The baseline model (model 1) only included adjustment for single year of age as a confounding variable, included as a second-order polynomial. We then introduced potential mediating factors sequentially, starting with factors associated with the risk of exposure to SARS-CoV-2 and then factors associated the risk of death if infected. Model 2 included additional adjustment for type of residence (private household, care home, or other communal establishments). In model 3, we included additional adjustment for geographical factors (region, Rural Urban classification and local population density). In model 4, we adjusted for socioeconomic and demographic factors that are likely to be linked to risk of infection (NS-SEC, highest qualification, IMD decile, household characteristics [tenure of the household, household deprivation, household size, family status, household composition, and key worker in the household], key worker type, individual and household exposure to disease, and individual and household proximity to others). We then adjusted for factors associated with the risk of death if infected. In model 5, additional adjustment was made for health status (pre-existing health conditions, BMI, and number of admissions to hospital and days spent in hospital over the previous 3 years). For all health variables, a binary interaction indicator was included, allowing the effects to vary depending on whether the individual was aged 70 years and older or younger than 70 years. In model 6, vaccination status (unvaccinated, one dose or two doses for wave two plus three doses for wave three) was included as a time-varying covariate, based on the date of vaccination plus 14 days. Therefore, an individual was classified as single-vaccinated 14 days after they received their first vaccine dose, double-vaccinated 14 days after they received their second dose, and triple-vaccinated 14 days after they received their third dose.

Missing Census data were imputed using nearest-neighbour donor imputation, the methodology employed by the Office for National Statistics across all 2011 Census variables [25]. Ethnicity was imputed in 3.0% of 2011 Census records due to item non-response. Individuals with missing data for BMI were placed into an unknown category. Health conditions were derived based on prescription and diagnosis codes, with the sample restricted to people who were registered with a GP practice in England that was participating in GDPPR. Therefore, there were no missing values.

All statistical analyses were stratified by sex and conducted using R, version 3.5. Cox proportional hazards models were implemented using the survival package (version 2.41-3) [26].

## Results

### Characteristics of the study population

The study population included 28,816,020 adults aged 30–100 years (mean age 55.8 years, SD 15.6) in England, 13,568,656 (47.1%) of whom were male (Table 1).

**Table 1:** Demographic and medical characteristics for the study cohort

Variable	Level	Study population
Age (years)	Mean (SD)	55.8 (15.6)
Sex	Male	13,568,656 (47.1)
	Female	15,247,364 (52.9)
Ethnicity	Bangladeshi	184,896 (0.6)
	Black African	397,661 (1.4)
	Black Caribbean	310,569 (1.1)
	Chinese	157,134 (0.5)
	Indian	785,099 (2.7)
	Mixed	343,007 (1.2)
	Pakistani	504,515 (1.8)
	White British^a^	23,932,242 (83.1)
	White other^b^	1,532,924 (5.3)
	Other^c^	667,973 (2.3)
Residence type	Private household	28,534,980 (99.0)
	Care home	163,681 (0.6)
	Other communal establishments	117,359 (0.4)
Region	North East	1,451,703 (5.0)
	North West	3,821,907 (13.3)
	Yorkshire and the Humber	2,922,921 (10.1)
	East Midlands	2,587,817 (9.0)
	West Midlands	3,001,415 (10.4)
	East	3,347,077 (11.6)
	London	3,725,727 (12.9)
	South East	4,871,486 (16.9)
	South West	3,085,967 (10.7)
Population density (people per km^2^)	Mean (SD)	4015.2 (4259.6)
Rural Urban classification	Major conurbation	9,169,912 (31.8)
	Minor conurbation	1,020,134 (3.5)
	City and town	12,743,541 (44.2)
	Town and fringe	2,889,540 (10.0)
	Village	1,861,972 (6.5)
	Hamlets and isolated dwellings	1,130,921 (3.9)
Index of Multiple Deprivation decile	1 (most deprived)	2,403,917 (8.3)
	2	2,536,450 (8.8)
	3	2,659,819 (9.2)
	4	2,799,824 (9.7)
	5	2,913,332 (10.1)
	6	3,037,189 (10.5)
	7	3,069,863 (10.7)
	8	3,117,088 (10.8)
	9	3,139,740 (10.9)
	10 (least deprived)	3,138,798 (10.9)
Highest qualification	No qualifications	5,662,228 (19.6)
	1–4 GCSEs/O-levels	3,997,818 (13.9)
	5+ GCSEs/O-levels	4,230,333 (14.7)
	Apprenticeship	1,047,989 (3.6)
	2+ A-levels or equivalent	3,422,513 (11.9)
	Degree or above	8,868,461 (30.8)
	Other qualification	1,586,678 (5.5)
National Statistics Socio-Economic Classification	Higher managerial occupations	3,271,478 (11.4)
	Lower managerial occupations	6,686,682 (23.2)
	Intermediate occupations	4,154,285 (14.4)
	Small employers and own account workers	2,960,524 (10.3)
	Lower supervisory and technical occupations	2,106,383 (7.3)
	Semi-routine occupations	4,268,923 (14.8)
	Routine occupations	3,280,595 (11.4)
	Never worked and long-term unemployed	1,439,862 (5.0)
	Not classified	647,288 (2.2)
Keyworker type	Not keyworker	22,954,450 (79.7)
	Education and childcare	1,775,302 (6.2)
	National and local government	222,672 (0.8)
	Public safety and national security	393,167 (1.4)
	Food and necessity goods	199,382 (0.7)
	Utilities and communication	382,723 (1.3)
	Transport	327,975 (1.1)
	Health and social care	2,107,948 (7.3)
	Key public services	452,401 (1.6)
Individual occupational proximity to others score	Mean (SD)	58.4 (20.1)
Individual occupational exposure to disease score	Mean (SD)	19.0 (21.0)
Household tenure	Owned outright	8,291,886 (28.8)
	Owned with mortgage	11,843,435 (41.1)
	Shared ownership	210,663 (0.7)
	Social rented from council	2,071,150 (7.2)
	Other social rented	1,783,495 (6.2)
	Private rented	4,078,886 (14.2)
	Living rent free	255,465 (0.9)
	Not in a household	281,040 (1.0)
Household deprivation	Not deprived	14,076,071 (48.8)
	Deprived in 1 dimension	8,899,172 (30.9)
	Deprived in 2 dimensions	4,200,396 (14.6)
	Deprived in 3 dimensions	1,237,718 (4.3)
	Deprived in 4 dimensions	121,623 (0.4)
	Not in a household	281,040 (1.0)
Household size	1 to 2 people	18,024,768 (62.6)
	3 to 4 people	9,305,381 (32.3)
	5 to 6 people	1079,374 (3.7)
	7+ people	125,457 (0.4)
	Not in a household	281,040 (1.0)
Family status	Not in a family	5,120,167 (17.8)
	In a couple family	20,634,579 (71.6)
	In a lone-parent family	2,780,234 (9.6)
	Not in a household	281,040 (1.0)
Household composition	Single-adult household	6,338,797 (22.0)
	Two-adult household	11,200,101 (38.9)
	Multi-generational household	2,199,382 (7.6)
	Other 3+ adults	3,233,029 (11.2)
	Child in household	5,563,671 (19.3)
	Not in a household	281,040 (1.0)
Keyworker in household	Yes	9,695,901 (33.6)
	No	18,839,079 (65.4)
	Not in a household	281,040 (1.0)
Maximum occupational proximity to others score in household	0 to < 20	560,696 (1.9)
	> 20 to < 40	192,508 (0.7)
	> 40 to < 60	9,238,006 (32.1)
	> 60 to < 80	12,517,686 (43.4)
	> 80 to < 100	6,026,084 (20.9)
	Not in a household	281,040 (1.0)
Maximum occupational exposure to disease score in household	0 to < 20	14,174,942 (49.2)
	> 20 to < 40	7,980,653 (27.7)
	> 40 to < 60	3,649,534 (12.7)
	> 60 to < 80	648,234 (2.2)
	> 80 to < 100	2,081,617 (7.2)
	Not in a household	281,040 (1.0)
Body mass index (kg/m^2^)	< 18.5	243,800 (0.8)
	18.5 to < 25.0	5,200,270 (18.0)
	25.0 to < 30.0	5,867,857 (20.4)
	> 30.0	5,078,348 (17.6)
	Unknown	12,425,745 (43.1)
Chronic Kidney disease	None	27,158,332 (94.2)
	Stage 3	1,519,711 (5.3)
	Stage 4	76,277 (0.3)
	Stage 5	61,700 (0.2)
Learning disability	None	28,641,212 (99.4)
	Learning disability	165,239 (0.6)
	Down syndrome	9569 (< 0.1)
Type 1 diabetes	None	28,525,099 (99.0)
	Type 1 diabetes with HbA_1c_< 59 mmol/mmol	117,414 (0.4)
	Type 1 diabetes with HbA_1c_ > 59 mmol/mmol	173,507 (0.6)
Type 2 diabetes	None	26,377,772 (91.5)
	Type 2 diabetes with HbA_1c_< 59 mmol/mmol	1,738,737 (6.0)
	Type 2 diabetes with HbA_1c_ > 59 mmol/mmol	699,511 (2.4)
Cancer and immunosuppression	Blood cancer	242,850 (0.8)
	Respiratory cancer	64,790 (0.2)
	Solid organ transplant	2896 (< 0.1)
Other health conditions	Asthma	3,312,344 (11.5)
	Atrial fibrillation	1,003,377 (3.5)
	Cerebral palsy	3623 (< 0.1)
	Chronic obstructive pulmonary disease	977,034 (3.4)
	Cirrhosis of the liver	74,871 (0.3)
	Congenital heart problem	78,510 (0.3)
	Congestive cardiac failure	476,097 (1.7)
	Coronary heart disease	1,456,798 (5.1)
	Dementia	325,589 (1.1)
	Epilepsy	320,981 (1.1)
	Osteoporotic fracture	25,958 (< 0.1)
	Parkinson’s disease	98,403 (0.3)
	Peripheral vascular disease	278,039 (1.0)
	Pulmonary hypertension or pulmonary fibrosis	106,305 (0.4)
	Rare pulmonary disease	338,294 (1.2)
	Rare neurological conditions	21,883 (< 0.1)
	Rheumatoid arthritis or systemic lupus erythematosus	299,442 (1.0)
	Severe combined immunodeficiency	12,739 (< 0.1)
	Severe mental illness	260,229 (0.9)
	Sickle cell disease	5327 (< 0.1)
	Stroke or transient ischaemic attack	795,197 (2.8)
	Venous thromboembolism	4298 (< 0.1)
Number of admissions to hospital in past 3 years	0	18,387,385 (63.8)
	1	5,195,755 (18.0)
	2 to 3	3,498,538 (12.1)
	4 to 5	965,541 (3.4)
	6 to 9	507,962 (1.8)
	10+	260,839 (0.9)
Number of days spent in hospital in past 3 years	0	25,383,455 (88.1)
	1	1,586,511 (5.5)
	2 to 4	1,306,820 (4.5)
	5 to 9	358,681 (1.2)
	10 to 19	119,435 (0.4)
	20 to 39	41,014 (0.1)
	40 to 69	12,368 (< 0.1)
	70+	7736 (< 0.1)

^a^White English/Welsh/Scottish/Northern Irish/British

^b^White Irish, White Gypsy or Irish Traveller, and other White

^c^Other Asian, Arab, other Black, and any other ethnic groups

**Fig. 2:**
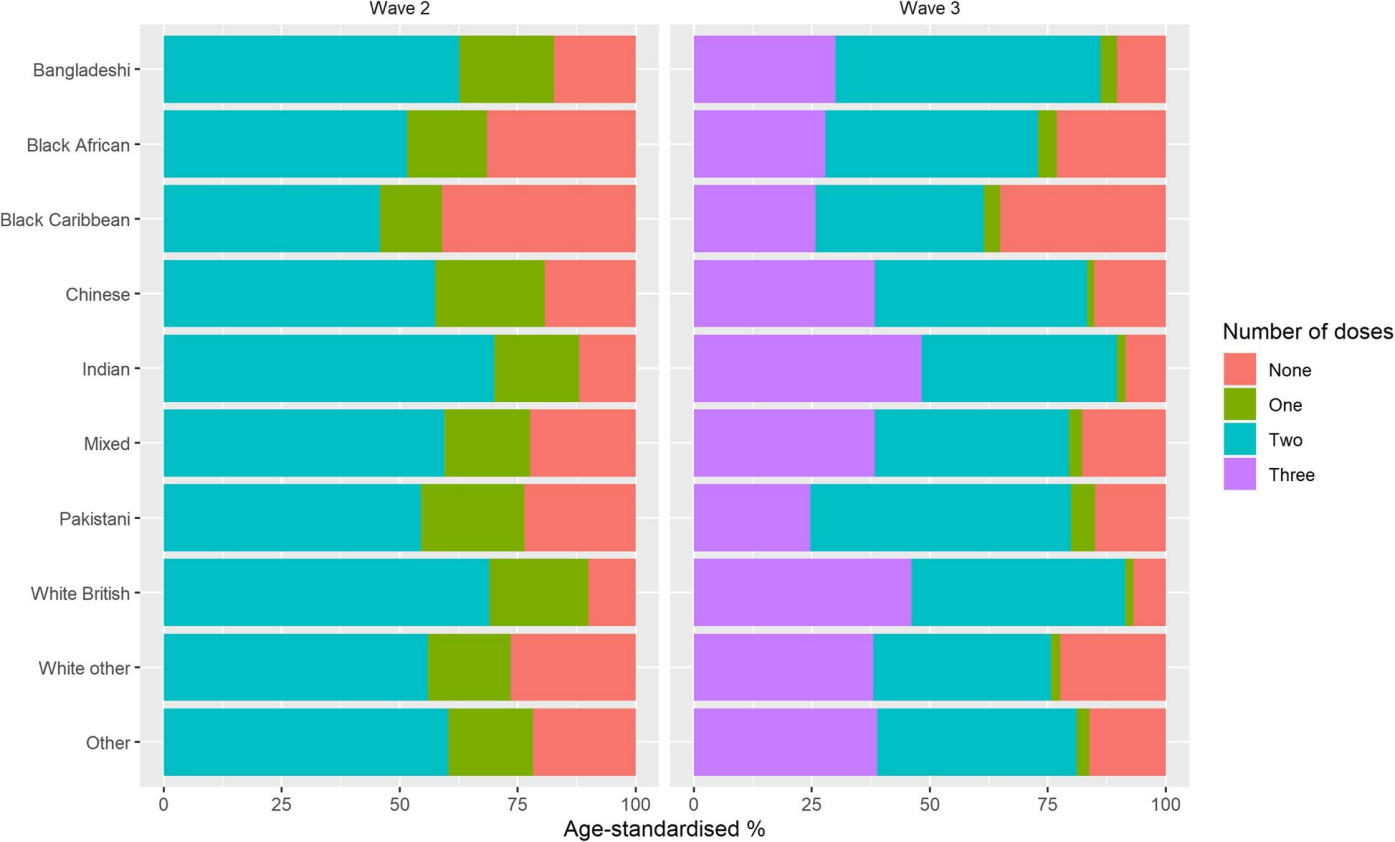
Age-standardised vaccination rates by ethnic group and wave of the pandemic. Percentage of people in each ethnic group that were unvaccinated or had received one or two doses of a COVID-19 vaccine by 12 June 2021 (left panel). Percentage of people in each ethnic group that were unvaccinated or had received one, two or three doses by 1 December 2021 (right panel)

446,093 deaths were recorded during follow-up, of which 66,558 were involving COVID-19 (54,770 in wave 2 and 11,788 in wave 3). Age-adjusted rates of receiving at least one COVID-19 vaccine dose by the end of the second wave (12 June 2021) were lowest among the Black Caribbean (41.0% unvaccinated) and Black African (31.5% unvaccinated) groups and highest among White British (10.1% unvaccinated) and Indian (12.0% unvaccinated) groups (Fig. 2). Ethnic inequalities in vaccination coverage continued into the third wave with the rollout of third/booster doses. As of 1 December 2021, age-adjusted rates of people receiving third/booster doses were lowest among the Pakistani group (24.6%) and highest for Indian (48.3%) and White British (46.1%) groups.

### Differences in COVID-19 mortality: age-standardised mortality rates

Overall, the ASMRs for death involving COVID-19 were higher than the White British group for all ethnic minority groups, except for the Chinese group and women in the White other group. Rates were highest for the Bangladeshi group and lowest for the Chinese group, men from the White British group, and women from the White other group (Table 2). Further disaggregating the ethnic groups showed that, compared with the White British group, ASMRs were elevated for the ‘other Asian’, ‘other Black’, and ‘any other’ ethnic groups and men from the Arab and White Gypsy or Irish Traveller groups (Additional file 1: Table S4). Breaking down the ASMRs by wave of the pandemic revealed that during both the second and third waves, the rate of death involving COVID-19 was consistently highest for the Bangladeshi group. Excess COVID-19 mortality relative to the White British group increased from the second to third wave for women from the Pakistani group and, to a lesser extent, women from the Bangladeshi and Black Caribbean group.

**Table 2:** Number of deaths and ASMRs (per 100,000 person-years) for deaths involving COVID-19, stratified by sex, ethnic group, and wave of the pandemic

	Total COVID-19 deaths		Wave 2 COVID-19 deaths		Wave 3 COVID-19 deaths	
	Count	ASMR (95% CI)	Count	ASMR (95% CI)	Count	ASMR (95% CI)
**Men**						
Bangladeshi	416	1283 (1134–1432)	337	1949 (1699–2200)	79	534 (397–696)
Black African	338	514 (437–592)	283	841 (704–978)	55	150 (95–217)
Black Caribbean	633	555 (508–602)	494	831 (752–910)	139	243 (199–287)
Chinese	105	292 (231–354)	87	473 (369–595)	18	89 (51–144)
Indian	1123	474 (443–505)	956	763 (710–817)	167	151 (125–176)
Mixed	219	384 (327–442)	180	597 (499–695)	39	145 (96–207)
Pakistani	885	776 (718–834)	680	1143 (1046–1239)	205	366 (307–425)
White British	29,998	291 (288–294)	24,250	445 (439–451)	5748	114 (111–117)
White other	1224	334 (314–354)	965	506 (472–540)	259	137 (119–155)
Other	696	443 (405–482)	569	697 (631–764)	127	159 (127–192)
**Women**						
Bangladeshi	269	669 (580–759)	212	1006 (854–1159)	57	294 (217–389)
Black African	232	258 (216–300)	170	364 (297–432)	62	141 (97–194)
Black Caribbean	523	322 (293–351)	384	449 (402–495)	139	180 (148–211)
Chinese	75	171 (131–218)	59	257 (191–339)	16	73 (40–121)
Indian	680	268 (247–289)	576	433 (396–470)	104	84 (67–101)
Mixed	191	259 (218–299)	156	408 (337–478)	35	90 (59–129)
Pakistani	530	449 (407–492)	386	625 (557–694)	144	254 (206–302)
White British	26,859	188 (186–191)	22,738	299 (295–303)	4121	62 (60–64)
White other	1079	179 (169–190)	895	282 (263–300)	184	63 (54–72)
Other	483	298 (268–328)	393	468 (417–520)	90	107 (82–135)

### Understanding the differences in COVID-19 mortality between ethnic groups

#### Second wave

**Fig. 3:**
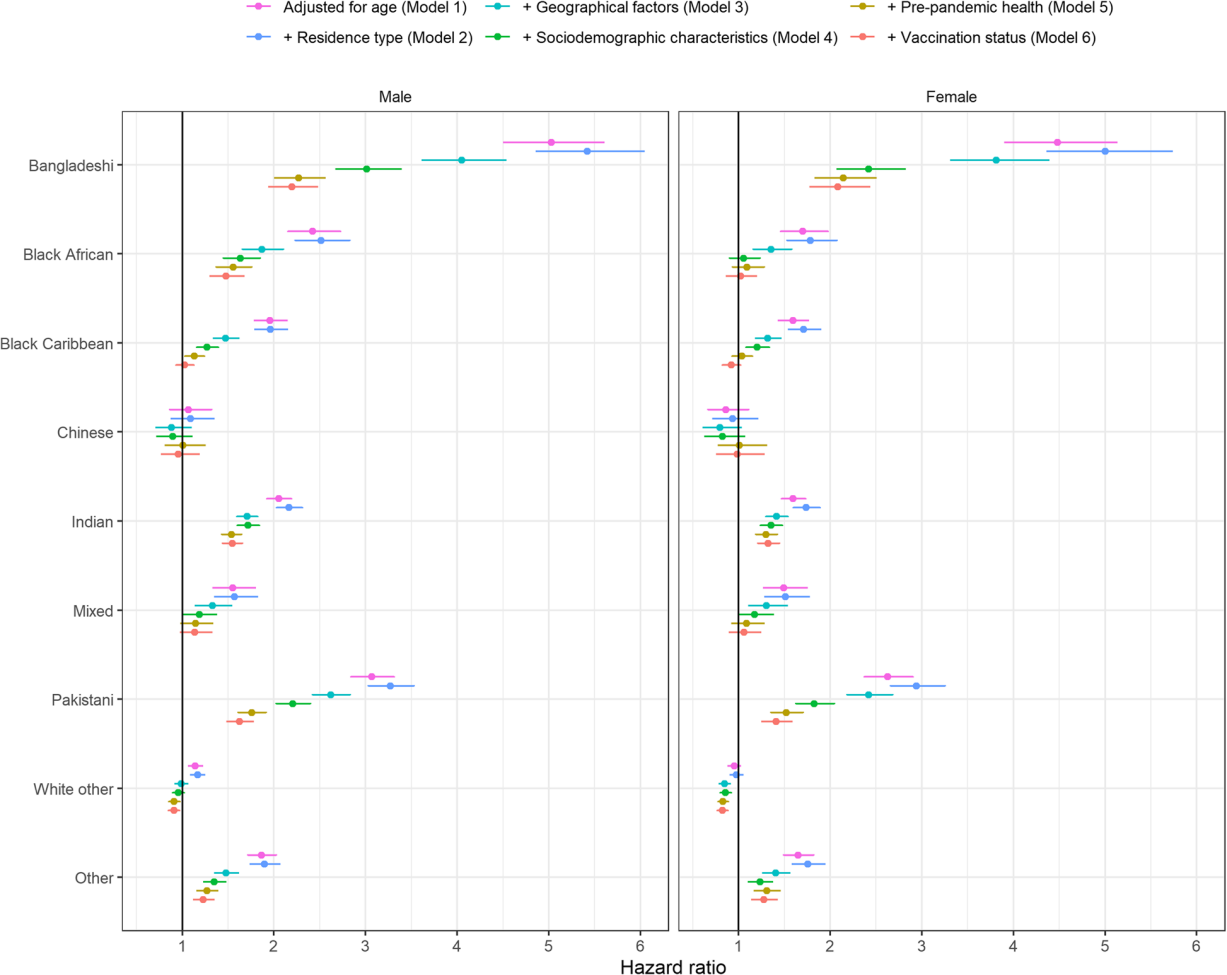
HRs for death involving COVID-19 by ethnic group during the second wave of the pandemic (8 December 2020 to 12 June 2021), relative to the White British group, stratified by sex. Results obtained from Cox proportional hazards regression models adjusted for the following: model 1—age; model 2—age plus residence type (private household, care home or other communal establishment); model 3—age and residence type plus geographical factors (region, Rural Urban classification and population density); model 4—age, residence type, and geography, plus sociodemographic factors (highest qualification, IMD decile, NS-SEC, household characteristics [tenure of the household, household deprivation, household size, family status, household composition and key worker in household], key worker type, individual and household exposure to disease, and individual and household proximity to others); model 5—age, residence type, geography, and sociodemographic factors, plus health status (pre-existing health conditions, BMI and hospital admissions over the previous 3 years); and model 6—age, residence type, geography, sociodemographic factors, and health status plus vaccination status (unvaccinated, one dose or two doses). Error bars represent 95% CIs

Age-adjusted hazard ratios (HRs) were calculated to examine the rate of death involving COVID-19 for ethnic minority groups relative to the White British group, holding the effect of age constant to account for the younger age distribution of ethnic minority groups. Compared with the White British group, rates of death involving COVID-19 were higher for all ethnic minority groups (except for the Chinese group and women from the ‘White other’ group) during the second wave (Fig. 3; model 1). HRs were highest for the Bangladeshi group, at 5.03 (95% confidence interval [CI] 4.51 to 5.60) for men and 4.48 (3.91 to 5.13) for women. HRs were substantially reduced after adjustment for geographical factors, socioeconomic status, and pre-existing health conditions (model 5) but remained elevated for the Bangladeshi (men: 2.27, 2.01 to 2.56; women: 2.14, 1.84 to 2.50), Pakistani (men: 1.76, 1.61 to 1.91; women: 1.52, 1.35 to 1.70), Indian (men: 1.53, 1.43 to 1.65; women: 1.30, 1.19 to 1.42), and ‘other’ (men: 1.27, 1.16 to 1.39; women: 1.31, 1.18 to 1.45) groups and for men from the Black African (1.55, 1.37 to 1.76) and Black Caribbean groups (1.13, 1.03 to 1.24). After further adjustment for vaccination status (model 6), the rate of death involving COVID-19 for men from the Black Caribbean and White British groups were similar. However, excess risk remained for the Bangladeshi (men: 2.20, 1.95 to 2.47; women: 2.08, 1.78 to 2.43), Pakistani (men: 1.62, 1.49 to 1.77; women: 1.41, 1.26 to 1.58), Indian (men: 1.54, 1.44 to 1.66; women: 1.32, 1.21 to 1.45), and ‘other’ (men: 1.23, 1.12 to 1.34; women: 1.28, 1.15 to 1.42) groups and for men from the Black African group (1.48, 1.30 to 1.67).

#### Third wave

**Fig. 4:**
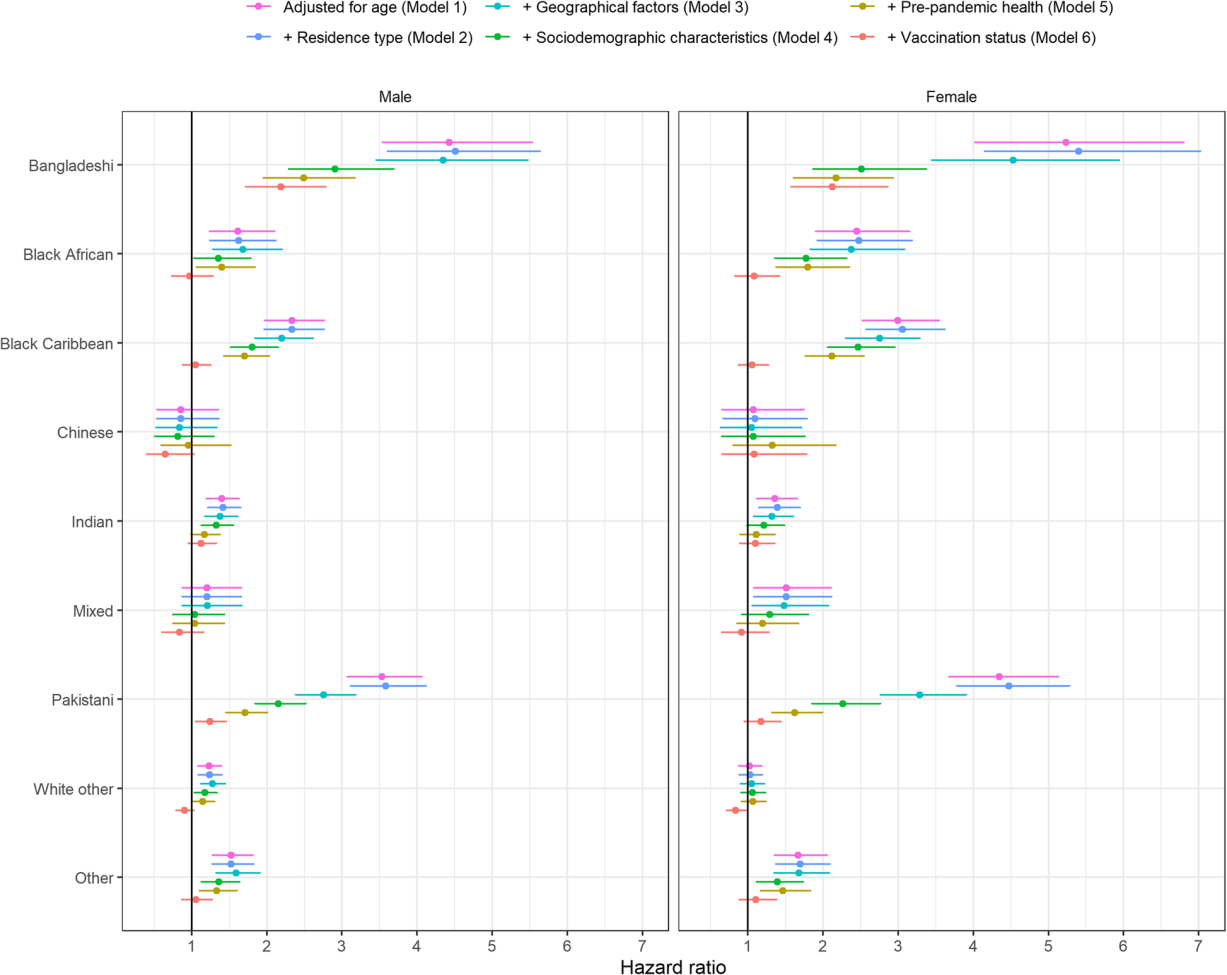
HRs for death involving COVID-19 by ethnic group during the third wave of the pandemic (13 June 2021 to 1 December 2021), relative to the White British group, stratified by sex. Results obtained from Cox proportional hazards regression models adjusted for the following: model 1—age; model 2—age plus residence type (private household, care home or other communal establishment); model 3—age and residence type plus geographical factors (region, Rural Urban classification and population density); model 4—age, residence type, and geography, plus sociodemographic factors (highest qualification, IMD decile, NS-SEC, household characteristics [tenure of the household, household deprivation, household size, family status, household composition and key worker in household], key worker type, individual and household exposure to disease, and individual and household proximity to others); model 5—age, residence type, geography, and sociodemographic factors, plus health status (pre-existing health conditions, BMI and hospital admissions over the previous 3 years); and model 6—age, residence type, geography, sociodemographic factors, and health status plus vaccination status (unvaccinated, one dose, two doses or third/booster dose). Error bars represent 95% CIs

During the third wave, age-adjusted HRs were elevated for all ethnic minority groups except for the Chinese group, men from the ‘mixed’ group, and women from the ‘White other’ group (Fig. 4, model 1). HRs continued to be highest for the Bangladeshi group (men: 4.43, 3.54 to 5.53; women: 5.23, 4.02 to 6.80). After adjusting for geographical factors, socioeconomic status, and pre-existing health conditions (model 5), HRs remained elevated for Bangladeshi (men: 2.49, 1.96 to 3.17; women: 2.17, 1.61 to 2.93), Pakistani (men: 1.71, 1.46 to 2.01; women: 1.62, 1.32 to 1.99), Black Caribbean (men: 1.70, 1.43 to 2.04; women: 2.12, 1.76 to 2.55), Black African (men: 1.40, 1.06 to 1.85; women: 1.80, 1.38 to 2.35), and ‘other’ groups (men: 1.33, 1.11 to 1.61; women: 1.46, 1.17 to 1.83) and men from the ‘White other’ group (1.15, 1.01 to 1.31). After additional adjustment for vaccination status (model 6), HRs remained elevated for the Bangladeshi group (men: 2.19, 1.72 to 2.78; women: 2.12, 1.58 to 2.86) and men from the Pakistani group (1.24, 1.06 to 1.46), whereas rates of death involving COVID-19 for all other groups were similar to the White British group.

## Discussion

Our findings demonstrate that throughout the rollout of the vaccine programme, most ethnic minority groups have continued to experience greater rates of death involving COVID-19 compared with the White British group. Although the patterns of excess COVID-19 mortality risk by ethnic group have changed over the course of the pandemic, the Bangladeshi, Black African, Black Caribbean, and Pakistani groups remained the groups with highest rate of COVID-19 mortality in the third wave. We also found that adjusting for vaccination status explained some of the remaining increased risk of COVID-19 mortality that is not explained by other factors, particularly during the third wave for the Black African, Black Caribbean, and Pakistani groups. After adjusting for geographical factors, sociodemographic characteristics, pre-pandemic health, and vaccination status, the risk of COVID-19 mortality was similar to the White British group for all ethnic groups except the Bangladeshi group and men from the Pakistani group.

Previous analyses showed that differences in location, measures of disadvantage, occupation, living arrangements, and certain pre-existing health conditions explain a large proportion (but not all) of the excess COVID-19 mortality risk observed in some ethnic groups [6, 7, 13]. Based on these findings, we hypothesised that ethnic differences in COVID-19 mortality could be mediated by factors associated with the risk of exposure to SARS-CoV-2 (e.g. geographical factors, socioeconomic status, living arrangements) and the risk of severe outcomes once infected (e.g. pre-existing health conditions, vaccination status) (Additional file 1: Fig. S2). We found that adjustment for geographical factors was associated with the largest reduction in the HRs for most ethnic groups during the second wave. SARS-CoV-2 case rates in England were higher in more densely populated urban areas than in less densely populated rural areas during the second wave [27]. Therefore, the reduction in risk of COVID-19 mortality in ethnic minority groups following adjustment for geographical factors likely reflects increased risk of infection. Additional adjustment for socioeconomic and demographic characteristics further reduced the HRs for most ethnic groups, most notably for Bangladeshi and Pakistani groups. This suggests that socioeconomic inequalities also contribute to increased rates of COVID-19 mortality in ethnic minority groups, which is consistent with evidence from other countries [2, 4, 28]. Further adjusting for pre-existing health conditions had a modest effect on the HRs for most ethnic groups, with the largest reductions for Bangladeshi and Pakistani men during the second wave. The smaller reduction in HRs for other ethnic groups possibly indicates that differences in the prevalence of comorbidities associated with severe COVID-19 outcomes were already captured by adjusting for socioeconomic inequalities that are also associated with health status.

Our results are consistent with studies that investigated ethnic inequalities in SARS-CoV-2 infection and COVID-19 mortality in the first two waves of the pandemic. There is ample evidence that most ethnic minority groups were disproportionately affected in the first two waves of the pandemic [5–7, 13, 29]. This study shows that the excess rate of death involving COVID-19 observed among ethnic minority groups early in the pandemic has continued throughout the rollout of the vaccine programme until late 2021. This is despite SARS-CoV-2 case rates being higher among the White British population from summer 2021 onwards [30, 31]. However, among people aged 65 years and over, cumulative case rates between March 2020 and October 2021 were higher for Black and South Asian groups than the White British group [32], suggesting that the age distribution of cases may partly explain the continued higher rates of COVID-19 mortality in these groups.

In addition, our findings suggest that lower vaccination rates (especially among Black Caribbean, Black African and Pakistani groups) contributes to explaining why some ethnic groups are more likely to experience more severe COVID-19 outcomes once infected. However, residual unexplained risk remained in the Bangladeshi group and men from the Pakistani group, even after full adjustment. People from Pakistani and Bangladeshi groups are more likely to reside in deprived areas, in large households and in multigenerational families [33]. Living in large, overcrowded, multi-generational households is associated with increased risk of SARS-CoV-2 infection [34, 35], and there is some evidence that living in a multi-generational household explains some of the differences in mortality [36]. Differences in occupational exposure may also account for some of the differences in mortality between groups, as a higher proportion of men from Pakistani and Bangladeshi groups work in key worker roles, such as healthcare workers, taxi drivers, shopkeepers, and proprietors than any other ethnic group [37], and these occupations have been found to be at elevated risk of COVID-19 mortality [38]. Whilst our study adjusted for a range of sociodemographic factors, including household composition and occupational exposure, these variables were retrieved from the 2011 Census, which may not reflect the situation in 2020 accurately. Consequently, adjustment for these factors might have been incomplete, possibly contributing towards the residual association.

The main strength of our study derives from using the ONS Public Health Data Asset, a nationwide large-scale population-wide data source combining the 2011 Census, mortality records, the General Practice Extraction Service (GPES) Data for Pandemic Planning and Research (GDPPR), Hospital Episode Statistics (HES), and vaccination data from the National Immunisation Management System (NIMS). Unlike studies based only on electronic health records, our study relies on self-identified ethnicity, limiting the potential for exposure misclassification bias. The PHDA also contains both detailed sociodemographic characteristics, such as household composition, housing quality, and occupational exposure, and extensive information on pre-pandemic health based on primary care and hospital records. To our knowledge, our study is the first to use nationally representative population-based linked data to examine the association between ethnicity and COVID-19 mortality in the third wave of the pandemic and explore the role of differences in vaccination uptake as a potential additional explanatory factor for the differences in COVID-19 mortality.

The main limitation is that most sociodemographic characteristics included in our models reflect the situations of individuals as they were in 2011, not necessarily those at the start of the COVID-19 pandemic. To mitigate this, we excluded people aged less than 30 years old, whose circumstances are the most likely to have changed since the Census. We also updated place of residence based on information from primary care records. As a result, information on area deprivation, rural/urban classification, region, and care home residence were up-to-date at the beginning of the pandemic. In addition, measurement error is likely to be smaller for the people at greater risk, since the sociodemographic factors are less likely to have changed for older people than younger people. However, some measurement error may reduce the explanatory power of the sociodemographic factors and pre-existing conditions included in the model, thus reducing their effect on the hazard ratios. For example, people who have retired since the 2011 Census will have incorrect data for current occupation. We have no reason to believe that there was substantial misdiagnosis of COVID-19 where it was mentioned on death certificates, especially in the latter part of the pandemic when testing was widely available.

Another limitation is that the study population is limited to people enumerated at the 2011 Census and therefore did not include people who were living in England but did not respond to the Census (estimated to be 5% of households) [39]. We also excluded people who did not link to the 2011 to 2013 National Health Service (NHS) Patient Registers. Whilst this does not affect the estimated association between ethnicity and COVID-19 mortality in our sample, it may affect the external validity of our study. Since the rate of linkage failure was higher for ethnic minority groups (with the highest rates of failure for the ‘other’, mixed and Chinese ethnic groups), the most likely result is an underestimation of the association between ethnicity and COVID-19 mortality in the population living in England at the beginning of the rollout of the COVID-19 vaccination programme, because a larger proportion of deaths occurring in ethnic minority groups will have been excluded. We sought to remove people who had emigrated since the 2011 Census from the population at-risk by restricting our study population to people registered with the NHS at the start of the pandemic. However, the study population also did not include people who immigrated or were born between 2011 and 2020. As a result, it did not fully represent the population at risk. Rates of immigration are higher for ethnic minority groups [40], which may mean that the denominator in our study population was less representative for some groups. In addition, deaths occurring among recent migrants would not have been included in our analyses. However, migrants overall tend to be young and the risk of COVID-19 mortality has been shown to be lower for young people [41]. Finally, single imputation using nearest-neighbour donor input for missing Census data may have inflated the statistical precision of Census variables.

Our study shows that adjusting for vaccination status eliminates the remaining elevated risk of COVID-19 mortality that is not explained by geographical factors, socioeconomic and demographic characteristics, and pre-existing health conditions in the third wave for Black African and Black Caribbean groups and reduces the remaining excess risk substantially for the Pakistani group (particularly for Pakistani women) compared to the White British group. The reduction in risk was more modest in the second wave, possibly reflecting widening ethnic inequalities in vaccination rates over time [10] and lower uptake of third vaccine doses during the third wave in some ethnic groups. As of 1 December 2021, the age-standardised rate of receiving three vaccine doses was 25.8% for the Black Caribbean group and 27.8% for the Black African group, compared to 46.1% for the White British group. Our results suggest that increasing vaccination uptake in ethnic minority groups could substantially reduce the inequalities in COVID-19 mortality. Strategies to increase vaccine uptake include improving trust in the efficacy and safety of vaccines by engaging with local community networks with lower vaccine uptake using appropriately tailored communication strategies. Identifying barriers to access, providing vaccinations in more convenient settings, and improving vaccine uptake in deprived areas could also increase vaccination rates in ethnic groups with low uptake [42, 43]. Future studies could also explore the potential impact that prioritisation for vaccination based on age, health conditions, and occupation may have had on long-term health outcomes (e.g. post-acute COVID-19 syndrome) in ethnic minority groups.

## Conclusions

The elevated rate of death involving COVID-19 observed among ethnic minority groups in the first phase of the pandemic has continued throughout the rollout of the vaccine programme until late 2021. Whilst much of these differences can be explained by geography, socioeconomic factors, and pre-existing health conditions, differences in COVID-19 vaccine coverage are also a key driver of the elevated risk of COVID-19 death, particularly among the Black African, Black Caribbean, and Pakistani groups. Increasing vaccine uptake in ethnic minority groups would help reduce the inequalities in COVID-19 mortality.

## Supplementary Information

**Additional file 1: Fig. S1.** Percentage of people with different postcodes in GP records and 2011 Census by age group. **Fig. S2.** Directed acyclic graph of the hypothesised relationship between ethnicity and COVID-19 mortality. **Table S1.** Rates and odds ratios of linkage failure between the 2011 Census and 2011 to 2013 NHS Patient Registers for people living in England at the 2011 Census who were aged 30-100 years in 2020, by sex, age group, ethnicity, region and IMD decile. **Table S2.** Coding and source of variables included in the analysis. **Table S3.** Weighted mean follow-up time in days by ethnic groups for wave two and wave three. **Table S4.** Age-standardised mortality rates (ASMRs) of death involving COVID-19 (8 December 2020 to 1 December 2021) for disaggregated ‘White other’ and ‘Other’ ethnic groups.

## Data Availability

Deidentified participant data and a data dictionary are available via the Secure Research Service for Accredited Researchers. For information on the Secure Research Service for Accredited Researchers see: https://www.ons.gov.uk/aboutus/whatwedo/statistics/requestingstatistics/approvedresearcherscheme. Analytical code is available on request to the corresponding author. As part of the Office for National Statistics wider research strategy, we are working towards making analytical code openly available.

## Abbreviations

ASMR: Age-standardised mortality rate
BMI: Body mass index
CI: Confidence interval
GDPPR: General Practice Extraction Service Data for Pandemic Planning and Research
GPES: General Practice Extraction Service
HES: Hospital Episode Statistics
HR: Hazard ratio
IMD: Index of Multiple Deprivation
NHS: National Health Service
NIMS: National Immunisation Management Service
NS-SEC: National Statistics Socio-economic Classification
ONS: Office for National Statistics
PHDA: Public Health Data Asset
SNOMED-CT: Systematized Nomenclature of Medicine Clinical Terms
SOC: Standard Occupational Classification
