# Supplementary material for: Ethnic differences in COVID-19 mortality in the second and third waves of the pandemic in England during the vaccine rollout: a retrospective, population-based cohort study
Source: BMC Med. 2023 Jan 8;21:13. doi: 10.1186/s12916-022-02704-7 (PMC9826727; doi:10.1186/s12916-022-02704-7)
Supplement: Supplementary file 1 — Additional file 1: Fig. S1. Percentage of people with different postcodes in GP records and 2011 Census by age group. Fig. S2. Directed acyclic graph of the hypothesised relationship between ethnicity and COVID-19 mortality. Table S1. Rates and odds ratios of linkage failure between the 2011 Census and 2011 to 2013 NHS Patient Registers for people living in England at the 2011 Census who were aged 30-100 years in 2020, by sex, age group, ethnicity, region and IMD decile. Table S2. Coding and source of variables included in the analysis. Table S3. Weighted mean follow-up time in days by ethnic groups for wave two and wave three. Table S4. Age-standardised mortality rates (ASMRs) of death involving COVID-19 (8 December 2020 to 1 December 2021) for disaggregated ‘White other’ and ‘Other’ ethnic groups. [file 12916_2022_2704_MOESM1_ESM.docx]

**Additional file 1**

**Fig. S1.** Percentage of people with different postcodes in GP records and 2011 Census by age group

**Fig. S2.** Directed acyclic graph of the hypothesised relationship between ethnicity and COVID-19 mortality

**Table S1.** Rates and odds ratios of linkage failure between the 2011 Census and 2011 to 2013 NHS Patient Registers for people living in England at the 2011 Census who were aged 30-100 years in 2020, by sex, age group, ethnicity, region and IMD decile

**Table S2.** Coding and source of variables included in the analysis

**Table S3.** Weighted mean follow-up time in days by ethnic groups for wave two and wave three

**Table S4.** Age-standardised mortality rates (ASMRs) of death involving COVID-19 (8 December 2020 to 1 December 2021) for disaggregated ‘White other’ and ‘Other’ ethnic groups

**Fig. S1.** Percentage of people with different postcodes in GP records and 2011 Census by age group


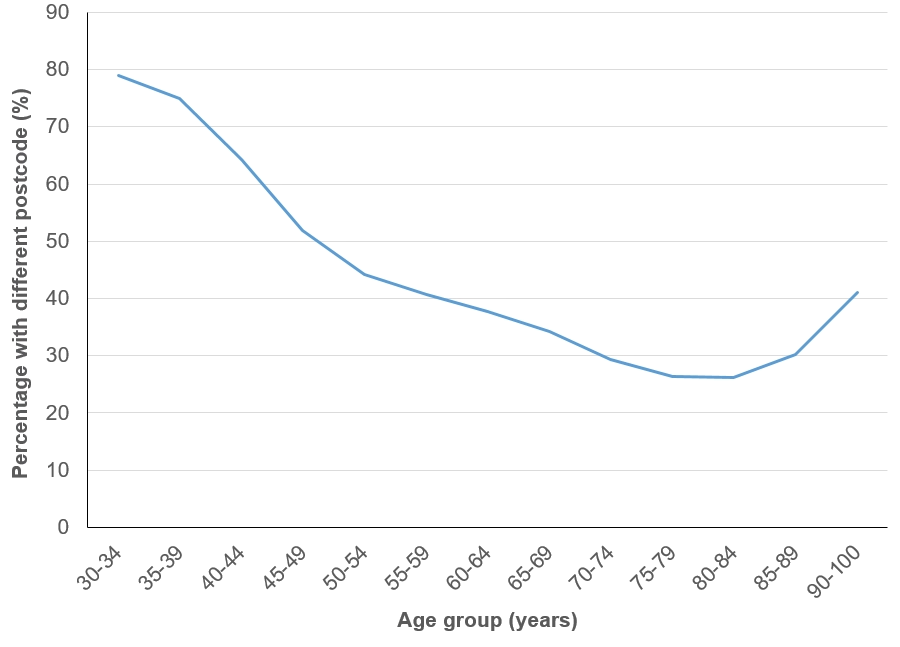


**Fig. S2.** Directed acyclic graph of the hypothesised relationship between ethnicity and COVID-19 mortality


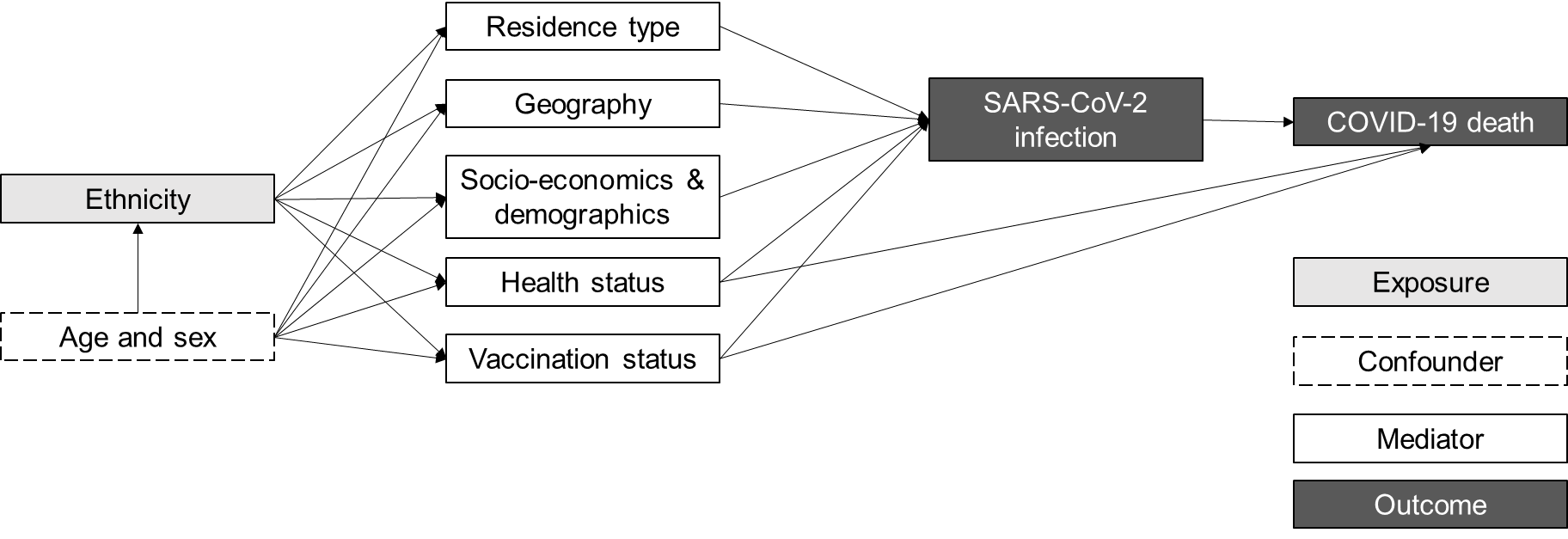


**Table S1.** Rates and odds ratios of linkage failure between the 2011 Census and 2011 to 2013 NHS Patient Registers for people living in England at the 2011 Census who were aged 30-100 years in 2020, by sex, age group, ethnicity, region and IMD decile

| **Characteristic** | **Level** | **N at 2011 Census** | **N linked to Patient Register** | **Rate of linkage failure (%)** | **Unadjusted OR (95% CI)** | **OR (95% CI) adjusted for age and sex** | **OR (95% CI) adjusted for age, sex, ethnicity, region and IMD decile** |
| --- | --- | --- | --- | --- | --- | --- | --- |
| Sex | Male | 19,082,910 | 17,794,510 | 6.8 | Ref | Ref | Ref |
|  | Female | 20,165,445 | 19,327,778 | 4.2 | 0.60 (0.60-0.60) | 0.61 (0.61-0.61) | 0.60 (0.60-0.60) |
| Age group (years) | 30-34 | 3,603,839 | 3,215,338 | 10.8 | 2.26 (2.24-2.27) | 2.26 (2.24-2.27) | 2.02 (2.01-2.03) |
|  | 35-39 | 3,669,872 | 3,280,872 | 10.6 | 2.21 (2.20-2.23) | 2.22 (2.21-2.23) | 1.93 (1.92-1.94) |
|  | 40-44 | 3,419,832 | 3,125,301 | 8.6 | 1.76 (1.75-1.77) | 1.76 (1.75-1.77) | 1.55 (1.54-1.56) |
|  | 45-49 | 3,672,722 | 3,432,183 | 6.5 | 1.31 (1.30-1.32) | 1.31 (1.30-1.32) | 1.24 (1.23-1.25) |
|  | 50-54 | 3,907,505 | 3,708,826 | 5.1 | Ref | Ref | Ref |
|  | 55-59 | 3,798,965 | 3,646,410 | 4.0 | 0.78 (0.78-0.79) | 0.78 (0.77-0.79) | 0.81 (0.80-0.81) |
|  | 60-64 | 3,269,023 | 3,165,585 | 3.2 | 0.61 (0.61-0.61) | 0.61 (0.60-0.61) | 0.65 (0.64-0.65) |
|  | 65-69 | 2,970,114 | 2,889,552 | 2.7 | 0.52 (0.52-0.52) | 0.52 (0.52-0.52) | 0.57 (0.57-0.58) |
|  | 70-74 | 3,092,578 | 3,018,843 | 2.4 | 0.46 (0.45-0.46) | 0.46 (0.45-0.46) | 0.52 (0.52-0.53) |
|  | 75-79 | 2,377,933 | 2,321,874 | 2.4 | 0.45 (0.45-0.46) | 0.45 (0.45-0.46) | 0.52 (0.52-0.53) |
|  | 80-84 | 1,990,717 | 1,942,031 | 2.4 | 0.47 (0.46-0.47) | 0.47 (0.47-0.48) | 0.54 (0.54-0.55) |
|  | 85-89 | 1,587,036 | 1,545,406 | 2.6 | 0.50 (0.50-0.51) | 0.51 (0.51-0.52) | 0.60 (0.59-0.60) |
|  | 90-100 | 1,888,219 | 1,830,067 | 3.1 | 0.59 (0.59-0.60) | 0.63 (0.62-0.64) | 0.74 (0.73-0.75) |
| Ethnicity | Bangladeshi | 245,164 | 226,755 | 7.5 | 1.86 (1.83-1.89) | 1.28 (1.26-1.30) | 0.92 (0.91-0.94) |
|  | Black African | 599,118 | 527,772 | 11.9 | 3.10 (3.07-3.12) | 2.36 (2.34-2.38) | 1.76 (1.74-1.77) |
|  | Black Caribbean | 445,503 | 413,411 | 7.2 | 1.78 (1.76-1.80) | 1.72 (1.70-1.74) | 1.30 (1.28-1.31) |
|  | Chinese | 286,419 | 242,791 | 15.2 | 4.11 (4.07-4.16) | 3.00 (2.97-3.03) | 2.69 (2.66-2.72) |
|  | Indian | 1,036,678 | 979,485 | 5.5 | 1.34 (1.33-1.35) | 1.05 (1.04-1.06) | 0.92 (0.92-0.93) |
|  | Mixed | 524,235 | 439,168 | 16.2 | 4.44 (4.40-4.47) | 3.20 (3.18-3.23) | 2.76 (2.73-2.78) |
|  | Pakistani | 650,738 | 610,439 | 6.2 | 1.51 (1.50-1.53) | 1.09 (1.08-1.10) | 0.84 (0.83-0.85) |
|  | White British | 31,923,883 | 30,587,948 | 4.2 | Ref | Ref | Ref |
|  | White other | 2,440,994 | 2,220,570 | 9.0 | 2.27 (2.26-2.28) | 1.78 (1.77-1.79) | 1.56 (1.55-1.57) |
|  | Other | 1,095,623 | 873,949 | 20.2 | 5.81 (5.78-5.84) | 4.54 (4.51-4.56) | 3.72 (3.70-3.74) |
| Region | North East | 1,940,553 | 1,845,711 | 4.9 | 1.09 (1.09-1.10) | 1.10 (1.09-1.11) | 0.95 (0.94-0.96) |
|  | North West | 5,214,200 | 4,928,347 | 5.5 | 1.24 (1.23-1.24) | 1.22 (1.22-1.23) | 1.03 (1.02-1.03) |
|  | Yorkshire & Humber | 3,886,983 | 3,672,437 | 5.5 | 1.24 (1.24-1.25) | 1.23 (1.22-1.23) | 1.06 (1.05-1.06) |
|  | East Midlands | 3,359,226 | 3,216,442 | 4.3 | 0.95 (0.94-0.95) | 0.95 (0.94-0.95) | 0.87 (0.86-0.87) |
|  | West Midlands | 4,096,396 | 3,888,547 | 5.1 | 1.14 (1.13-1.15) | 1.12 (1.12-1.13) | 0.93 (0.92-0.93) |
|  | East | 4,349,516 | 4,161,689 | 4.3 | 0.96 (0.96-0.97) | 0.96 (0.96-0.97) | 0.95 (0.94-0.95) |
|  | London | 6,025,721 | 5,470,154 | 9.2 | 2.16 (2.15-2.17) | 1.84 (1.83-1.85) | 1.18 (1.17-1.18) |
|  | South East | 6,389,962 | 6,103,433 | 4.5 | Ref | Ref | Ref |
|  | South West | 3,985,798 | 3,835,528 | 3.8 | 0.83 (0.83-0.84) | 0.86 (0.86-0.87) | 0.84 (0.84-0.85) |
| IMD decile | 1 (most deprived) | 3,695,758 | 3,359,470 | 9.1 | 3.43 (3.40-3.45) | 2.83 (2.81-2.85) | 2.45 (2.43-2.46) |
|  | 2 | 3,829,820 | 3,515,392 | 8.2 | 3.06 (3.04-3.08) | 2.50 (2.48-2.52) | 2.07 (2.06-2.09) |
|  | 3 | 3,917,708 | 3,638,070 | 7.1 | 2.63 (2.61-2.65) | 2.20 (2.19-2.22) | 1.87 (1.86-1.88) |
|  | 4 | 3,956,957 | 3,712,222 | 6.2 | 2.26 (2.24-2.27) | 1.95 (1.93-1.96) | 1.73 (1.72-1.74) |
|  | 5 | 4,010,325 | 3,798,473 | 5.3 | 1.91 (1.89-1.92) | 1.71 (1.70-1.72) | 1.58 (1.56-1.59) |
|  | 6 | 4,017,641 | 3,830,053 | 4.7 | 1.68 (1.66-1.69) | 1.56 (1.55-1.57) | 1.46 (1.45-1.47) |
|  | 7 | 4,011,304 | 3,846,311 | 4.1 | 1.47 (1.46-1.48) | 1.41 (1.40-1.42) | 1.36 (1.34-1.37) |
|  | 8 | 3,988,411 | 3,840,555 | 3.7 | 1.32 (1.31-1.33) | 1.28 (1.27-1.29) | 1.25 (1.24-1.26) |
|  | 9 | 3,956,310 | 3,827,347 | 3.3 | 1.15 (1.14-1.16) | 1.13 (1.12-1.14) | 1.12 (1.12-1.13) |
|  | 10 (least deprived) | 3,864,121 | 3,754,395 | 2.8 | Ref | Ref | Ref |

**Table S2.** Coding and source of variables included in the analysis

| **Variable** | **Coding** | **Source(s)** |
| --- | --- | --- |
| Ethnicity | Bangladeshi, Black African, Black Caribbean, Chinese, Indian, Mixed, Pakistani, White British, White Other, Other | 2011 Census |
| Age | Single year of age (second-order polynomial) | 2011 Census |
| Sex | Male, female | 2011 Census |
| Residence type | Private household, care home, other communal establishments | 2011 Census and 2019 NHS Patient register |
| Region | North East, North West, Yorkshire and the Humber, East Midlands, West Midlands, East, London, South East, South West | Postcodes from GDPPR and National Statistics Postcode Lookup (November 2019) |
| Population density | Second-order polynomial, allowing for a different slope beyond the 99^th^ percentile to account for extreme values | Postcodes from GDPPR and mid-year 2019 population estimates |
| Rural Urban classification | Major conurbation, minor conurbation, city and town, town and fringe, village, hamlets and isolated dwellings | Postcodes from GDPPR and National Statistics Postcode Lookup (November 2019) |
| Index of Multiple Deprivation | Dummy variables representing deciles of deprivation | Postcodes from GDPPR and English Indices of Deprivation, 2019 |
| Highest qualification | No qualifications, 1-4 GCSEs/O-levels, 5+ GCSEs/O-levels, apprenticeship, 2+ A-levels or equivalent, degree or above, other qualification | 2011 Census |
| National Statistics Socio-Economic Classification | Higher managerial occupations, lower managerial occupations, intermediate occupations, small employers and own account workers, lower supervisory and technical occupations, semi-routine occupations, routine occupations, never worked and long-term unemployed, not classified | 2011 Census |
| Keyworker type | Not keyworker, education and childcare, national and local Government, public safety and national security, food and necessity goods, utilities and communications, transport, health and social care, key public services | 2011 Census |
| Individual occupational proximity to others score | Score ranging from 0 (do not work near other people) to 100 (work very close to other people) | 2011 Census and O*NET database |
| Individual exposure to disease score | Score ranging from 0 (no exposure) to 100 (maximum exposure) | 2011 Census and O*NET database |
| Household tenure | Owned outright, owned with mortgage, shared ownership, social rented from council, other social rented, private rented, living rent free, not in a household | 2011 Census |
| Household deprivation | Not deprived, deprived in 1 dimension, deprived in 2 dimensions, deprived in 3 dimensions, deprived in 4 dimensions, not in a household | 2011 Census |
| Household size | 1 to 2 people, 3 to 4 people, 5 to 6 people, 7+ people, not in a household | 2011 Census |
| Family status | Not in a family, in a couple family, in a lone-parent family, not in a household | 2011 Census |
| Household composition | Single-adult household, two-adult household, multi-generational household, other 3+ adults, child in household, not in a household | 2011 Census |
| Keyworker in household | Yes, no, not in a household | 2011 Census |
| Maximum occupational proximity to others score in household | 0 to < 20, > 20 to < 40, > 40 to < 60, > 60 to < 80, > 60 to < 80, not in a household | 2011 Census |
| Maximum occupational exposure to disease score in household | 0 to < 20, > 20 to < 40, > 40 to < 60, > 60 to < 80, > 60 to < 80, not in a household | 2011 Census |
| Body mass index (kg/m^2^) | < 18.5, 18.5 to < 25.0, 25.0 to < 30.0, > 30.0, Unknown | GDPPR |
| Chronic kidney disease | None, stage 3, stage 4, stage 5 | GDPPR |
| Learning disability | None, learning disability, Down syndrome | GDPPR |
| Type 1 diabetes | None, Type 1 diabetes with HbA_1c_ < 59mmol/mmol, Type 1 diabetes with HbA_1c_ > 59mmol/mmol | GDPPR |
| Type 2 diabetes | None, Type 2 diabetes with HbA_1c_ < 59mmol/mmol, Type 2 diabetes with HbA_1c_ > 59mmol/mmol | GDPPR |
| Cancer and immunosuppression | Binary flags for blood cancer, respiratory cancer, solid organ transplant | GDPPR |
| Other health conditions | Binary flags for asthma, atrial fibrillation, cerebral palsy, chronic obstructive pulmonary disease, cirrhosis of the liver, congenital heart problem, congestive cardiac failure, coronary heart disease, dementia, epilepsy, osteoporotic fracture, Parkinson’s disease, peripheral vascular disease, pulmonary hypertension or pulmonary fibrosis, rare pulmonary disease, rare neurological conditions, rheumatoid arthritis or systemic lupus erythematosus, severe combined immunodeficiency, severe mental illness, sickle cell disease, stroke or transient ischaemic attack, venous thromboembolism | GDPPR |
| Number of admissions to hospital in past 3 years | 0, 1, 2 to 3, 4 to 5, 6 to 9, 10+ | HES APC |
| Number of days spent in hospital in past 3 years | 0, 1, 2 to 4, 5 to 9, 10 to 19, 20 to 39, 40 to 69, 70+ | HES APC |
| Vaccination status | Unvaccinated, one dose, two doses, three doses (time-varying based on vaccination date lagged by 14 days) | NIMS |

GDPPR, General Practice Extraction Service Data for Pandemic Planning and Research; HES APC, Hospital Episode Statistics Admitted Patient Care; NIMS, National Immunisation Management Service

**Table S3.** Weighted mean follow-up time in days by ethnic groups for wave two and wave three

| **Ethnic group** | **Weighted mean follow-up time (days)** | |
| --- | --- | --- |
|  | **Wave 2** | **Wave 3** |
| Bangladeshi | 185.3 | 170.8 |
| Black African | 185.7 | 170.8 |
| Black Caribbean | 185.0 | 170.5 |
| Chinese | 185.7 | 170.8 |
| Indian | 185.4 | 170.7 |
| Mixed | 185.6 | 170.8 |
| Pakistani | 185.5 | 170.7 |
| White British | 185.0 | 170.4 |
| White other | 185.4 | 170.7 |
| Other | 185.6 | 170.8 |

**Table S4.** Age-standardised mortality rates (ASMRs) of death involving COVID-19 (8 December 2020 to 1 December 2021) for disaggregated ‘White other’ and ‘Other’ ethnic groups

|  | **Total deaths involving COVID-19** | |
| --- | --- | --- |
|  | Count | ASMR (95% CI) |
| **Men** |  |  |
| Arab | 78 | 421 (319-541) |
| Other Asian | 384 | 456 (401-511) |
| Other Black | 85 | 454 (341-588) |
| White Irish | 497 | 317 (287-348) |
| White Gypsy or Irish Traveller | 39 | 654 (412-961) |
| White other | 688 | 336 (309-363) |
| Any other ethnic group | 149 | 420 (344-496) |
|  |  |  |
| **Women** |  |  |
| Arab | 30 | 242 (153-360) |
| Other Asian | 269 | 298 (257-338) |
| Other Black | 76 | 371 (281-479) |
| White Irish | 483 | 184 (167-201) |
| White Gypsy or Irish Traveller | 19 | 325 (166-550) |
| White other | 577 | 171 (157-186) |
| Any other ethnic group | 108 | 283 (226-341) |
